# Notch2, a Key Player in Chronic Lymphocytic Leukemia: Mechanism, Microenvironment Interactions, and Therapeutic Implications

**DOI:** 10.3390/cancers18030518

**Published:** 2026-02-05

**Authors:** Ramona Miserendino, Claudio Giacinto Atene, Mario Luppi, Roberto Marasca, Stefania Fiorcari

**Affiliations:** 1 Hematology Unit, Department of Oncology and Hematology, Azienda-Ospedaliero Universitaria (AOU) of Modena, Policlinico, 41124 Modena, Italy; 2 Department of Medical and Surgical Sciences, Section of Hematology, University of Modena and Reggio Emilia, 41124 Modena, Italy; 3 Transfusion Medicine Unit, Department of Oncology and Hematology, Azienda-Ospedaliero Universitaria (AOU) of Modena, Policlinico, 41124 Modena, Italy

**Keywords:** chronic lymphocytic leukemia, Notch2, tumor microenvironment, drug resistance, tissue niche, survival, B cell proliferation, target agents, signaling pathway

## Abstract

**Background/Objectives**: Tissue niches, such as those in the spleen, bone marrow, and lymph nodes, are crucial for the survival and growth of leukemic cells in chronic lymphocytic leukemia (CLL). **Methods**: A growing amount of research over the last 20 years has shown how important the tumor microenvironment (TME) is to the pathophysiology, development, and resistance to treatment of CLL. This protective environment, which is made up of various cell types (including stromal and immune cells), extracellular matrix components, and soluble factors, supports CLL cells and encourages their survival, growth, and drug resistance. Even in the absence of mutations, Notch2 is functionally activated in the CLL system, in addition to the well-known Notch1. This occurs because leukemic cells aberrantly express the ligand Jagged1/2, which activates the Notch2 receptor on both stromal and CLL cells themselves. Notch2 activation on stromal cells leads to the triggering of the Wnt/β-catenin program in CLL cells, whereas the activation of Notch2 in CLL cells promotes the expression of Mcl-1, which confers drug tolerance (especially in cases with trisomy 12). **Results**: In addition to these mechanisms, Notch2 acts as a transcription factor that directly controls the expression of key targets, such as CD23 and Hes1, that are fundamental for B cell proliferation, differentiation, and survival in CLL. **Conclusions**: All of these circuits represent important therapeutic targets and help explain the cells’ dependence on their niche, the formation of proliferation centers, and resistance to modern targeted agents.

## Simple Summary

Chronic lymphocytic leukemia cells depend on the protective “niches” within the body to grow and resist treatments. This supportive system is orchestrated by Notch2. Even without genetic mutations, Notch2 becomes overactive because the leukemia background releases continuous signals that turn it on. This activation creates a vicious cycle. When Notch2 is triggered on surrounding support cells, they send growth signals back to the leukemia cells. Simultaneously, when triggered on the leukemia cells themselves, Notch2 produces proteins that block cell death and encourage proliferation. This dual mechanism explains why the leukemic clone is so hard to eliminate with standard drugs. Understanding this communication network reveals crucial targets for therapy aimed at disrupting this vicious cycle.

## 1. Chronic Lymphocytic Leukemia and Notch Family

With an incidence of roughly 4.5 new cases per 100,000 people each year, chronic lymphocytic leukemia (CLL) is the most prevalent adult leukemia in Western countries. Even while CLL is a generally indolent disease in its early stages, it can develop into a more aggressive form (such as Richter transformation) and frequently shows resistance to common chemotherapies. Although the exact cause of this clinical heterogeneity is as of yet unknown, genetic factors and the intricate interactions that leukemic cells exhibit with their surroundings are the key determinants. Among the critical cytogenetic abnormalities that influence disease course, trisomy 12 is a recurring event, found in approximately 10% to 20% of CLL cases [1,2,3]. Patients with this abnormality are often characterized by accelerated disease progression, significant lymph node involvement, and a greater frequency of Notch1 gene mutations. The Notch signaling pathway is a highly conserved intercellular communication system that controls basic biological functions such as apoptosis, proliferation, and cell-fate determination. In humans, four transmembrane receptors mediate this pathway: Notch1, Notch2, Notch3, and Notch4. When its extracellular domain is bound by an exogenous ligand (Delta-like or Jagged protein) the classical activation begins. Enzymes like TACE and the gamma-secretase complex enhance a series of proteolytic cleavages of the Notch receptor that are triggered by this interaction. The release of the Notch intracellular domain (NICD) is the final and most crucial step. After that, the NICD fragment translocates to the nucleus, where it combines with other co-activators and the DNA-binding protein CSL to create a complex. In its basal state, CSL acts as a transcriptional repressor. However, the complex switches from a repressor to a strong transcriptional activator when NICD binds to it. This leads to the transcription of target genes that support cell survival, proliferation, and drug resistance [4].

## 2. The Physio-Pathological Role of Notch2 in B Cell Development and Differentiation

The NOTCH2 gene encodes Notch2, a crucial member of the Notch receptor family that carries out non-redundant functions in adult tissue maintenance and development. Notch2 plays a critical role in the immune system’s marginal zone B cell specification. This specific role in normal B lymphocyte differentiation provides the key biological rationale for its frequent dysregulation in B cell malignancies, such as CLL and splenic marginal zone lymphoma (SMZL), which often co-opt these physiological developmental pathways for survival and proliferation [5,6]. In CLL cells, both Notch1 and Notch2 receptors are found to be constitutively activated, proving to be a consistent feature. Unlike NOTCH1 gene mutations, which are observed in CLL and are associated with poor prognosis and Richter transformation, mutations in the NOTCH2 gene are notably rare or absent in CLL [7]. Of note, multiple mechanisms contribute to determining NOTCH1 pathway dysregulation, driving the expansion of CLL clones. In particular, NOTCH1 mutations are found in up to 20% of CLL patients and are related to protein truncation, loss of the PEST domain, and accumulation of NICD, resulting in the upregulation of Nocth1 signaling [8,9,10]. Despite this lack of genetic alterations, NOTCH2 is transcriptionally active, and its pathway is critically involved in maintaining the malignant phenotype of CLL lymphocytes. Moreover, continuous activation of Notch2 is often observed in patients with trisomy 12, providing a significant survival advantage. Indeed, high levels of Notch2 enable CLL cells to resist programmed cell death and proliferate uncontrollably. In fact, the resistance to apoptosis is linked to the activation of anti-apoptotic proteins such as Mcl-1. As a consequence, the augmented expression of Mcl-1 correlates with a poor prognosis for CLL patients [11,12]. Similarly, another critical target gene of Notch2 is cMyc, a proto-oncogene that encodes transcription factors that are essential for fundamental cellular processes, such as cell cycle progression, growth, and apoptosis. It is estimated that Myc can directly or indirectly regulate up to 15% of all genes in the human genome [13]. In addition to its role within the cell, cMyc controls and mediates various biological effects through immune system cells and the tumor microenvironment. Indeed, when the MYC gene is overexpressed, it can become an oncogene, leading to its overexpression in many cancer settings, in approximately 30% of human cancers [14].

## 3. The Microenvironment’s Influence in CLL

CLL cells are highly dependent on their surrounding microenvironment. Inside lymphoid tissues, B CLL cells are in close contact with the stromal environment, and their subsequent cell interactions play a key role in the development of the disease [15]. CLL cells are able to circulate between the bloodstream and secondary lymphoid organs and vice versa. This traffic is enabled by the secretion of certain soluble factors, such as chemokines (IL-4, IL-10) and growth factors, like VEGF and FGF. The homing process depends on fine adjustments between chemokines and ligands, secreted by stromal cells, and their receptors present on B CLL cells [16]. Furthermore, CLL cells increase their migratory capacity and tissue homing thanks to the presence of the extracellular matrix, which provides a scaffold for their movements.

### Components of the CLL Microenvironment

• *Stromal Cells:* The CLL tumor microenvironment is composed of various stromal components, including fibroblasts, endothelial cells, mesenchymal stem cells (MSCs), and nurse-like cells (NLCs). The latter two, in particular, are crucial for CLL pathogenesis, because in lymphatic tissue, they create a protective niche that nurtures leukemic B cells, providing CLL cells survival and proliferation stimuli through a variety of mechanisms [1]. Through direct contact, stromal cells provide survival stimuli to CLL cells, which thus leave the lymphatic organs and enter the bloodstream. These factors include growth factors, chemokines, and cytokines (e.g., IL-4, IL-6, TNF-a, CCL3, CCL4, CXCL12), which help to keep the leukemic clone alive in the lymph nodes and bone marrow. These factors can activate key signaling pathways like NF-kB and PI3K/AKT, which are central to cancer cell survival. In addition, stromal cells also communicate with CLL cells through extracellular vesicles that are exocytosed from the cells. These vesicles transport molecules that enter the leukemic cell and act to modify their genetic expression and behavior, activating or repressing the expression of specific genes. Conversely, CLL cells can also act by influencing the tumor microenvironment [17], producing vesicles containing proteins and microRNAs that in turn can reprogram stromal cells, turning them into cancer-associated fibroblasts (CAFs). These CAFs, in addition, secrete a variety of factors that further support leukemia clones [18]. Finally, TME also mediates the drug resistance that leukemic cells develop, partly due to contact with stromal cells. In fact, when CLL cells are in close contact with stromal cells, they tend to be less sensitive to traditional treatments, such as fludarabine, and also develop resistance to target agents, such as venetoclax, a well-known Bcl-2 inhibit. Stromal cells enable leukemic cells to resist drugs by upregulating the expression of anti-apoptotic factors such as Mcl-1 [19,20]. In summary, the TME shapes disease outcomes, and it is not merely a passive bystander but an active participant in CLL pathogenesis. Indeed, it affects cell survival, proliferation, migration, and drug resistance, making TME a crucial therapeutic target. Therefore, the general characteristics of cancer networks are also present in CLL: leukemic cells are shielded from immune destruction, growth factors are generated locally, and new vessels supply nutrients [21].
• *Extracellular Matrix (ECM):* Inside the lymphoid tissues, the ECM supports leukemic cells by providing stimuli that induce survival and tissue infiltration. Alterations in ECM components, including fibronectin, collagen, and laminin, along with other molecules like proteoglycans, are commonly observed in CLL. These components make available adhesion sites for CLL cells and stromal cells, and this provides anti-apoptotic signals that protect cells from death and activate drug resistance mechanisms. In addition, CLL cells together with stromal cells secrete enzymes like matrix metalloproteinases (MMPs) that break down and remodel the ECM, facilitating migration and providing additional cell attachment sites to promote a protective environment within the niches of bone marrow and lymph nodes [22]. The resulting remodeling also releases growth factors that further fuel the survival and proliferation of cancer cells [23]. In particular, CLL cells bind the ECM through receptors such as Cd44, leading to the activation of pro-survival pathways such as PI3K/Akt, with consequent induction of survival proteins like Mcl-1 [24]
• *Immune Cells**:* The TME is heavily populated by immune cells that can both suppress immunovigilance mechanisms and directly affect CLL cells by activating their survival mechanisms. Under normal conditions, T cells are responsible for killing cancer cells, but in CLL, this function appears to be compromised. In CLL patients, T cells become exhausted due to chronic exposure to leukemic cells, resulting in a reduced ability to form functioning immune “synapses”. Furthermore, the Treg population is highly abundant and exerts immunosuppressive effects on T cells, thereby inhibiting their anti-tumor immune response. This creates a state of immune tolerance that effectively shields CLL clones. One of the most important axes, and among the most studied in CLL, is the CD40L/CD40 axis. This interaction is provided by CD4+ T cells, which supply CD40L to CLL cells that receive the signal via the CD40 receptor, through which important proliferation signals are mediated, protecting CLL cells from programmed death [25]. Equally important players in the immune system in CLL are macrophages and their precursors, monocytes, which participate in the TME and cooperate with leukemic cells. Specifically, CLL cells induce monocytes to differentiate into an immunosuppressive, M2-like macrophage phenotype, called nurse like cells in the context of CLL. These cells secrete factors that promote tumor growth and suppress the anti-tumor immune response and are called tumor-associated macrophages (TAMs). NLCs release chemokines such as CXCL12 and CXCL13, which are able to attract and protect CLL cells from spontaneous and drug-induced apoptosis [26,27]. Furthermore, by presenting antigens to other immune cells in an ineffective manner, macrophages may also contribute to immune evasion and weaken the overall response against leukemia. Lastly, natural killer (NK) cells are part of the innate system and are normally responsible for killing cancer cells. Despite an increase in their number, in CLL, this ability is compromised. Indeed, their ability to kill cancer cells in this context is reduced due to decreased expression of activating receptors and increased expression of inhibitory markers. Furthermore, CLL cells are able to suppress NK cells themselves through different mechanisms such as the production of immunosuppressive cytokines like TGF-beta, BAG6, and HLA-G, leading to the inhibition of NK cell activity and affecting the “killing signals” [28]. In addition, CLL cells escape from NK cells’ control by altering actin cytoskeleton remodeling, with consequent resistance to NK cell cytotoxicity [29].

## 4. Notch2 and Tumor Microenvironment Interaction in CLL

In the tissue microenvironment, Notch signaling is involved in cellular crosstalk through cell-to-cell contact. In particular, the complex interaction between stromal and immune cells enhances the activation of Notch2 signaling in cancer cells, stimulating an immunosuppressive milieu. These interconnections support cancer growth, the evasion of immune surveillance, and resistance to treatment. In this context, the mechanisms involved in stimulating Notch2 activation are related to juxtacrine signaling and paracrine loops [30]. The interplay between Notch2-mediated signals and the TME is central to the development and progression of CLL. In a mutualistic manner, CLL cells take advantage of Notch2 to manipulate the surrounding microenvironment, which produces signals that in turn activate the Notch2 signaling pathway, creating a positive feedback loop that promotes cell survival, proliferation, and resistance to therapies by leukemia cells. One of the fundamental exchanges between TME and CLL cells is mediated by the glycoprotein CD23, which is expressed by both B cells and T cells. CD23 regulates the proliferation of normal B and T cells and human IgE synthesis. The binding of T cell-derived B cell growth factors (BCGFs), such as IL-4, IL-2, or TGF-β, to B cells produces a cleaved soluble form of CD23 [31]. The soluble form is abundant in the serum of CLL patients, and it has been demonstrated that the intracellular domain of Notch2 participates in the transcriptional complex that induces the overexpression of CD23 in CLL cells [32]. Another peculiar feature in CLL is the expression of Notch2 ligands, Jagged1 and Jagged2 [33]. These ligands bind to Notch receptors present on TME cells, such as MSCs and NLCs, triggering the production of factors in stromal cells that support leukemic cells. This induces the Wnt signaling pathway, a key pro-survival pathway. When Notch2 is activated in MSCs, the production of C1q and other soluble factors is upregulated. These factors bind to the Frizzled receptor on the surface of CLL cells, mimicking canonical Wnt ligands. This process activates WNT/β-catenin signaling in the leukemic clones, resulting in increased survival and resistance to apoptosis. The aforementioned reciprocal signaling loop is one of the hallmarks within the malignant niche in CLL [34,35]. Furthermore, the intricate relationship between Notch2 and the TME also has significant consequences for drug resistance: high Notch2 expression, in particular in CLL patients with trisomy 12, is linked to elevated levels of Mcl-1: one of the most important anti-apoptotic members of the Bcl-2 family. The induction of Mcl-1 confers less sensitivity to venetoclax in CLL cells, a commonly used BCL-2 inhibitor and currently the most effective target agent for the treatment of CLL [11]. Thereby, together, the Wnt and Mcl-1 pathways are crucial for CLL pathogenesis and therapeutic resistance, particularly as they are often activated by signals from the tumor microenvironment. Lastly, in the TME, Notch signaling regulates TAM function through modulation of HES-1 expression. HES-1 expression is upregulated in TAM, supporting a peculiar influence on immunosuppression mediation. Indeed, the expression of HES-1 in the TAM population exacerbates their immunosuppressive profile, in particular affecting T cell function. This could play a major role in mediating the immunosuppression mechanisms that occur in the CLL niche [36]. All of the above-mentioned signaling pathways contribute to the survival of leukemic clones, particularly within secondary lymphoid organs, allowing them to form a leukemia reservoir that is resistant to treatment.

### 4.1. The Notch2/CD23 Axis in CLL Cell Survival and Homeostasis

**Figure 1:**
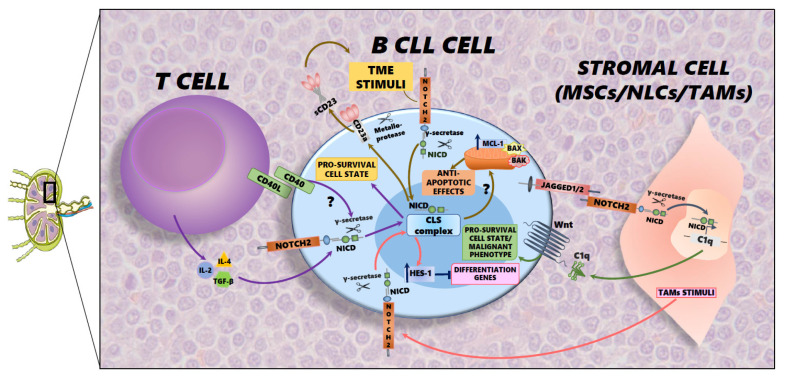
Schematic representation of the main interaction involving Notch2 signaling in B CLL cells within the tumor microenvironment. On the left is a T cell that, through the release of chemokines and the exposure of the CD40 ligand on the membrane, stimulates the Notch2 receptor on the leukemic clone. The intracellular domain (NICD) is activated and, migrating to the nucleus, binds the CLS complex, activating the transcription of genes involved in cell survival. On the right, stromal cells, for example, TAMs, release soluble stimuli activating the Notch2 receptor on B CLL cells. This initiates the transcription of the HES1 gene, which blocks the transcription of differentiation genes. Alternatively, MSCs, which express the Notch2 receptor in their membrane, are stimulated by the Jagged1/2 ligand exposed on the leukemic cell. This interaction activates intracellular signaling in the stromal cell, leading to the transcription of the soluble factor C1q. C1q is released into the extracellular tumor microenvironment and binds the Wnt receptor expressed by the CLL cell. In the leukemic cell, Wnt triggers the transcription of genes, such as Cyclin D1 and cMYC, that contribute to the formation of a malignant phenotype. In the middle, stimuli from the tumor microenvironment activate the signaling of Notch2 expressed on the membrane of the CLL cell, and it acts as a transcription factor within the nucleus, activating the transcription of CD23a. At the cytoplasmatic membrane level, through the action of metalloproteases, CD23a is released into the extracellular matrix in its soluble form, triggering pro-proliferative signaling in the TEM. In addition, the same microenvironmental factors stimulate Notch2 on B cells, which increases, through an unknown mechanism, the expression of Mcl-1. Mcl-1, sequestering the Bax and Bad proteins, acts as an anti-apoptotic factor, resulting in the survival of the tumor cell.

One of the most important features of CLL is the overexpression of CD23, a transmembrane glycoprotein that has been recognized as an important marker of B cell differentiation/activation. CD23 acts as the receptor for IgE and it consists of two isoforms, CD23a and CD23b, and the expression of CD23a is specifically linked to B lymphocytes. In particular, the overexpression of the CD23a isoform has been correlated to CLL cell survival [32] and its soluble form, cleaved by metalloproteinase, is released into the microenvironment, contributing to the clinical burden of the disease. Some studies have demonstrated that the intracellular domain of Notch2 is found to be a part of the transcription complex that binds the responsive element of CD23a in the core promoter region in CLL cells. Thereby, this evidence directly links the constitutive activation of Notch2 within CLL cells and the constant overexpression of CD23 in those same cells [32]. An additional mechanism of the Notch2–CD23 axis is also mediated by PKCδ. In fact, its activation makes CLL cells resistant to γ-secretase inhibitors. The inhibition of PKCδ, on the other hand, downregulates Notch2 expression and the resulting pro-proliferative activity mediated by CD23. Therefore, PKCδ stimulation leads to activation of the Notch2–CD23 cascade in CLL cells [37]. The sustained expression of CD23, in turn, is thought to promote the stability and trafficking of CLL cells, contributing to the maintenance of the malignant clone both in the circulation and within protective tissue niches. This Notch2-mediated link to CD23 expression highlights another mechanism by which this receptor, even in the absence of canonical mutations, drives the malignant phenotype and potentially affects the clinical course of CLL (Figure 1).

### 4.2. Notch2-Driven Activation of Wnt Signaling: A Pro-Survival Pathway

The Wnt signaling pathway is an essential cellular communication system that governs fundamental processes like cell proliferation, differentiation, and survival. In adult mammals, Wnt is also an important regulator of the majority of tissue stem cell types, and cancer and several growth-related disorders are caused by mutant Wnt pathway components [38]. The activation of the canonical Wnt/β-catenin pathway in CLL cells is often not a result of intrinsic mutations but is instead non-autonomous, being dictated by the tumor microenvironment via Notch2 crosstalk [34]. This aberrant activation entails the switching from a developmental controller to a key oncogenic driver. In the absence of an activating Wnt signal, β-catenin is continuously phosphorylated for its destruction. This tagging marks the protein for recognition by ubiquitin and subsequent degradation by the proteasome, keeping β-catenin levels low and the pathway transcriptionally inactive. Instead, the activation of the pathway begins when Wnt ligands bind to their corresponding transmembrane receptors on the cell surface. The activated receptors recruit and inhibit the “destruction complex”. With its inhibitors disabled, β-catenin escapes phosphorylation and subsequent degradation, leading to its rapid accumulation in the cytoplasm. The accumulated β-catenin then translocates into the nucleus, and here it acts as a transcriptional co-activator partnering with members of the TCF/LEF (T cell factor/lymphoid enhancer-binding factor) family of DNA-binding transcription factors. This association switches the TCF/LEF complex from its repressor form to that of a transcriptional activator, leading to the expression of a number of pro-survival genes, such as c-MYC and Cyclin D1, which promote a malignant phenotype [39]. This mechanism effectively links the supportive stromal cells directly to the CLL cell’s proliferative machinery. From the other side, tumor cells stimulate Notch2 activity in stromal cells, inducing the transcription of complement factor C1q. C1q is able to inhibit the GSk3β pathway in leukemic cells. C1q can activate the Wnt co-receptor LRP5/6, inducing β-catenin stabilization [34].

### 4.3. Notch2-Mediated Upregulation of Mcl-1: The Anti-Apoptotic Guardian

Notable studies have linked the action of Notch2 to the anti-apoptotic phenotype that B cells assume in CLL, particularly in cases with trisomy 12. In fact, regardless of the Wnt pathway, Notch2 supports cell survival through the expression of Mcl-1 [11]. Mcl-1 (Myeloid cell leukemia sequence-1) is one of the most important members of the Bcl-2 family, which orchestrates programmed cell death processes. This mechanism is mediated by different factors that, when in conflict with each other, can lead to apoptosis or cell survival. The anti-apoptotic members, like Bcl-2, Mcl-1, and Bcl-xL, sequester the pro-apoptotic proteins, such as Bax and Bak, preventing them from triggering the usual programmed death mechanism. Conversely, when Bax and Bak are discharged, they can act freely, creating pores in the outer mitochondrial membrane through which cytochrome C is released, thus initiating the caspase-mediated cascade that leads to cell death. In particular, Mcl-1 prevents pore formation, sequestering these proteins, thereby maintaining mitochondrial integrity and ensuring cell survival [40]. High levels of Mcl-1 have been documented in various forms of cancer, as well as in CLL. Specifically, studies involving the use of specific Bcl-2 inhibitors, such as venetoclax, highlight an overexpression of Mcl-1, which, through a compensatory mechanism following the inhibition of Bcl-2, triggers secondary anti-apoptotic mechanisms. In fact, venetoclax acts by covalently binding Bcl-2, which thus stops sequestering the pro-apoptotic members that can initiate the cell death cascade. However, in these contexts, Mcl-1 is overexpressed by malignant cells, bypassing the inhibition of the target agent. In this way, Mcl-1 remains free to sequester Bax and Bak, triggering an alternative pathway of cell survival [41]. This compensatory mechanism has been described most extensively in CLL patients with trisomy 12, representing a more aggressive subtype of the disease, directly dependent on the chromosomal abnormality or on signals from the tumor microenvironment mediated by Notch2. In fact, in primary CLL samples with trisomy 12 in which Notch2 has been silenced, Mcl-1 expression is consequently reduced; similarly, treating trisomy 12 samples with specific Mcl-1 inhibitors restores sensitivity to venetoclax [11].

### 4.4. Hes1 and Notch2: Mediating the Niche Survival Signal in CLL

The oncogenic role of Notch2 signaling in CLL is also executed through the induction of one of its canonical downstream targets, the transcription factor Hairy and Enhancer of Split 1 (Hes1). Hes1 acts as a critical mediator of the proliferative and survival signals received by CLL cells from the TME. Indeed, as demonstrated in several studies, it has emerged that all B cell patient samples express Hes1, which instead is undetectable in PBMCs of healthy donors [33]. When Notch2 is stimulated by interaction with its ligands, supplied by T cells or stromal cells present in the microenvironment, it initiates the transcription of Hes1, which in turn controls the transcription of genes involved in the anti-apoptotic pathway and the cell cycle. In particular, Hes1 is a key repressor of cell differentiation, and this mechanism is mediated by the upregulation of antiapoptotic factors, such as Mcl-1, which sustains cell survival, and by the overexpression of adhesion molecules, which reinforce tumor cells’ dependence on their niche. Indeed, this mechanism has been described in certain hematological malignancies in which Hes1 acts as an oncogene by blocking cell differentiation through the repression of C/EBP-α and promoting a stem-like phenotype in leukemia cells [42]. Therefore, Hes1 contributes as an additional mediator of the Notch2 pathway in communications between leukemia cells and the tumor microenvironment, participating in the complex machinery of drug resistance in CLL.

## 5. Conclusions: The Notch2-TME Axis as the Engine of CLL Drug Resistance

The pathogenesis and progression of CLL are driven by intricate communication between leukemic B cells and the tumor microenvironment, which is fundamentally orchestrated by Notch2. This complex relationship underlies resistance to modern target agents, such as venetoclax. Nevertheless, a deep understanding of this crucial interaction can provide new therapeutic possibilities that are not only aimed at targeting leukemic cells, but also at supporting microenvironment. Acting directly on the axis of interaction between CLL cells and tumor niche cells, by blocking the Notch2 signaling pathway, could offer new perspectives for patients, allowing for the elimination of the apoptosis-resistant clone and enabling them to achieve complete remission from the disease. Various strategies can be pursued to target Notch2: (1) act directly on the cleavage of Notch2 on its intracellular portion, using γ-secretase inhibitors (GSIs) to block its migration within the nucleus and consequent activation as a transcription factor, preventing the transcription of its genetic targets, such as Mcl-1 and Wnt [43]; (2) use GSIs in combination with existing target agents to block both the Bcl-2-mediated anti-apoptotic pathway and BCR signaling, thereby simultaneously disabling TME signals and eliminating CLL cells; (3) use anti-tumor immune strategies (e.g., checkpoint inhibitors) that can synergize with anti-Notch2 agents to increase efficacy and reverse the immune evasion implemented by the microenvironment. The rationale behind these combined strategies is supported by evidence that the Notch pathway influences the expression of immune checkpoints, such as PD-1/PD-L1, and modulates the immunosuppressive TME signals [44] (Table 1). As suggested by several studies, activation of the Notch pathway is involved in drug resistance profiles. Different combination therapies may overcome resistance through targeting compensatory signaling pathways such as Mcl-1, PI3K, and NF-kB. In addition, since the Notch pathway promotes immune evasion, increasing PD-L1 and immunosuppressive cells, the possibility of targeting Notch may impact the immunosuppressive milieu by increasing T cell infiltration and boosting PD-1/PD-L1 inhibition [45]. Overall, these strategies aim to synergistically overcome the survival advantage that the TME confers to CLL by dismantling both the direct support provided by the tumor niche and the anti-apoptotic mechanisms of leukemic cells, both potentially mediated by Notch2.

## Figures and Tables

**Table 1:** Schematization of three therapeutic strategies for treating chronic lymphocytic leukemia by targeting the Notch2 pathway.

Clinical Goal	Molecular Target	Mechanism of Action	Drug Type/ Approach	Strategy
Block Notch2 migration to the nucleus and prevent transcription of survival genes (Mcl-1, Wnt).	Notch2, Mcl-1, Wnt	Acts directly on the cleavage of the intracellular portion of Notch2.	γ-secretase inhibitors (GSIs)	*Direct Inhibition*
Disable TME support and eliminate apoptosis-resistant CLL cells.	Notch2, Bcl-2, BCR	Simultaneous blockade of Bcl-2/BCR pathways and tumor microenvironment signals.	GSIs + existing target agents (e.g., Venetoclax or BCR inhibitors)	2. *Combined Therapy*
Enhance immune efficacy by modulating immunosuppressive signals regulated by Notch2.	Notch2, PD1, PD-L1	Synergistic action to reverse immune evasion mediated by the microenvironment.	Anti-Notch2+ checkpoint inhibitors (e.g., PD1/PD-L1 inhibitors)	3. *Synergistic Immunotherapy*

## Data Availability

No new data were created or analyzed in this study.

## Abbreviations

The following abbreviations are used in this manuscript:

CLL	Chronic Lymphocytic Leukemia
TME	Tumor Microenvironment
NICD	Notch Intracellular Domain
SMZL	Splenic Marginal Zone Lymphoma
MSCs	Mesenchymal Stem Cells
NLCs	Nurse-Like Cells
CAFs	Cancer-Associated Fibroblasts
ECM	Extracellular Matrix
MMPs	Matrix Metalloproteinases
TAMs	Tumor-Associated Macrophages
NK	Natural Killer
BCGFs	B Cell Growth Factors
TCF	T Cell Factor
LEF	Lymphoid Enhancer-binding Factor
Mcl-1	Myeloid cell leukemia sequence-1
Hes1	Hairy and Enhancer of Split 1
GSIs	γ-Secretase Inhibitors
